# Assessment of Common Cyanotoxins in Cyanobacteria of Biological Loess Crusts

**DOI:** 10.3390/toxins14030215

**Published:** 2022-03-16

**Authors:** Tamara Dulić, Zorica Svirčev, Tamara Palanački Malešević, Elisabeth J. Faassen, Henna Savela, Qingzhen Hao, Jussi Meriluoto

**Affiliations:** 1 Department of Biochemistry, Faculty of Sciences and Engineering, Åbo Akademi University, Tykistökatu 6A, 20520 Turku, Finland; zorica.svircev@dbe.uns.ac.rs (Z.S.); jussi.meriluoto@abo.fi (J.M.); 2 University of Novi Sad, Faculty of Sciences, Department of Biology and Ecology, Trg Dositeja Obradovića 3, 21000 Novi Sad, Serbia; tamara.palanacki@dbe.uns.ac.rs; 3 Wageningen Food Safety Research, Wageningen University and Research, Akkermaalsbos 2, 6708 WB Wageningen, The Netherlands; els.faassen@wur.nl; 4 Aquatic Ecology and Water Quality Management, Wageningen University and Research, Droevendaalsesteeg 3a, 6708 PB Wageningen, The Netherlands; 5 Department of Life Technologies, Faculty of Technology, University of Turku, Kiinamyllynkatu 10, 20014 Turku, Finland; henna.savela@utu.fi; 6 Laboratory of Cenozoic Geology and Environment, Institute of Geology and Geophysics, Chinese Academy of Sciences, No. 19, Beitucheng Western Road, Beijing 100029, China; haoqz@mail.iggcas.ac.cn

**Keywords:** cyanotoxins, biocrusts, loess, terrestrial cyanobacteria, sedimentary biosignatures, land restoration

## Abstract

Cyanotoxins are a diverse group of bioactive compounds produced by cyanobacteria that have adverse effects on human and animal health. While the phenomenon of cyanotoxin production in aquatic environments is well studied, research on cyanotoxins in terrestrial environments, where cyanobacteria abundantly occur in biocrusts, is still in its infancy. Here, we investigated the potential cyanotoxin production in cyanobacteria-dominated biological loess crusts (BLCs) from three different regions (China, Iran, and Serbia) and in cyanobacterial cultures isolated from the BLCs. The presence of cyanotoxins microcystins, cylindrospermopsin, saxitoxins, and β-N-methylamino-L-alanine was analyzed by liquid chromatography-tandem mass spectrometry (LC-MS/MS) method, while the presence of cyanotoxin-encoding genes (*mcyE*, *cyrJ*, *sxtA*, *sxtG*, *sxtS*, and *anaC*) was investigated by polymerase chain reaction (PCR) method. We could not detect any of the targeted cyanotoxins in the biocrusts or the cyanobacterial cultures, nor could we amplify any cyanotoxin-encoding genes in the cyanobacterial strains. The results are discussed in terms of the biological role of cyanotoxins, the application of cyanobacteria in land restoration programs, and the use of cyanotoxins as biosignatures of cyanobacterial populations in loess research. The article highlights the need to extend the field of research on cyanobacteria and cyanotoxin production to terrestrial environments.

## 1. Introduction

Cyanobacteria produce various bioactive metabolites, some of which exert toxic effects on human and animal cells and cause chronic and acute diseases. Cyanotoxins can be classified in two ways, based on their chemical structure and their main toxic [1]. With more than 200 known congeners [2], microcystins (MCs) are the most commonly detected cyanotoxins worldwide, followed by cylindrospermopsins (CYNs), anatoxins (ATXs), and saxitoxins (STXs), while the most frequently reported toxic cyanobacterial strains belong to genera *Microcystis*, *Dolichospermum* (prev. *Anabaena)*, *Aphanizomenon*, *Planktothrix*, and *Oscillatoria* [3].

Some observed routes of exposure to cyanotoxins are drinking water, aquatic recreational activities, dialysis, and food items (contaminated fish, shellfish, and agricultural and horticultural products) [4]. More than 180 cases of cyanotoxin poisoning in humans and animals have been reported to date, mainly associated with toxic cyanobacterial blooms [3]. Partly for this reason, the focus of cyanotoxin research has been primarily on cyanobacterial bloom events and toxin production by aquatic cyanobacteria. Although Prinsep et al. [5] and Honkanen et al. [6] reported MC in cultures of cyanobacteria isolated from terrestrial environments, including moist and garden soils and wet rocks, little attention has been paid to cyanotoxin production in arid and semi-arid terrestrial environments, in which cyanobacteria abundantly occur within biocrusts. So far, cyanotoxins microcystin and apratoxin were detected in strains isolated from biocrusts [7]. Metcalf et al. [8] reported the presence of MC-LR and *mcy D* genes in cyanobacterial biocrusts in the deserts of Qatar, while several other studies [9,10,11] reported the presence of β-N-methyl-amino-L-alanine BMAA and its isomers DAB and N-(2-aminoethyl)glycine (AEG) in the same region (please note the concerns raised about the methodology of the BMAA analysis [12,13]).

Biocrusts are an important functional unit in drylands, representing an association of sediment/soil particles and various organisms—bacteria, cyanobacteria, algae, fungi, microfauna, lichens, and bryophytes. Their micromorphology and species composition vary spatially and temporally. Cyanobacteria in biocrusts provide important ecological services, including soil surface stabilization through immobilization and aggregation of particles, carbon and nitrogen cycling and storage, and control of infiltration and runoff [14,15,16]. Contribution of cyanobacteria to the global biocrust cover is substantial ([17], Figure 9.4), as they are found on each of the continents on a range of sediment types from the finest silt to the coarsest sand. Therefore, it is not surprising that they are also found extensively on exposed surfaces of loess deposits and sandy soils in deserts.

Loess deposits are formed by the accumulation of windblown dust particles (60–90% silt) consisting mainly of quartz, feldspar, and mica and cover more than 10% of the land surface, and have been extensively studied as one of the most important paleoclimate archives in the semi-arid and arid regions. These are polygenic deposits characterized by complex sedimentation and post-depositional processes known as loessification [18]. The latest hypothesis on loess formation in arid and semi-arid regions, the BLOCDUST hypothesis, has recognized cyanobacteria in biological loess crusts (BLC) as a significant contributor to this process through the accumulation and stabilization of aeolian dust particles [19,20].

A suite of biomarkers and biomarker proxies, mainly related to higher plant community composition [21], are commonly used to interpret the paleoclimate and paleoenvironment of loess deposits. Bacteria-derived biomarkers, namely branched glycerol dialkyl glycerol tetraethers (brGDGTs), have been used to reconstruct air temperature and pH in loess [22,23]. In addition, Li et al. [24] and Shen et al. [25] observed a bimodal n-alkane distribution between C_14_ and C_33_, indicating a significant contribution of a microbial community to the signal. These results could be in favor of the BLOCDUST hypothesis, so it is crucial to develop further cyanobacteria-specific biosignatures as tools in loess research.

Loess deposits, because of their porous structure and extensive exploitation and degradation, are a significant source of airborne dust affecting people living in loess-rich areas. With a similar idea to the BLOCDUST hypothesis on the interaction between cyanobacteria and loess particles, artificial formation of BLC cover over the degraded loess surfaces by inoculation of cyanobacteria is proposed as a promising mechanism for their restoration [26,27]. The technology is based on the inoculation of laboratory-grown cyanobacteria on disturbed soil surfaces to establish soil particle stability, nutrient uptake, and circulation, and promote the establishment of higher vegetation [28,29,30]. It is, therefore, important that the cyanobacterial strains used for inoculation do not express toxicity to humans and animals, because particles of the crust containing potentially toxic cyanobacteria could become airborne and affect human and animal health by inhalation [1,8].

The toxicity of cyanobacterial taxa within BLCs has only recently been addressed for the first time. In the two studies, one BLC sample [31] and one cyanobacterial strain isolated from a BLC [27] showed low toxicity to the *Artemia salina* larvae, indicating the necessity for further studies. This study aims to investigate cyanotoxin production in cyanobacteria-dominated BLCs and in cyanobacterial strains isolated from BLCs to address the following: 1. the extent of cyanotoxin production in cyanobacteria of BLC; 2. to avoid the application of potentially toxic strains in the restoration of deteriorated loess surfaces; 3. potential of cyanotoxins as a biosignature of cyanobacterial communities in paleoenvironmental research of loess.

## 2. Results

**Figure 1:**
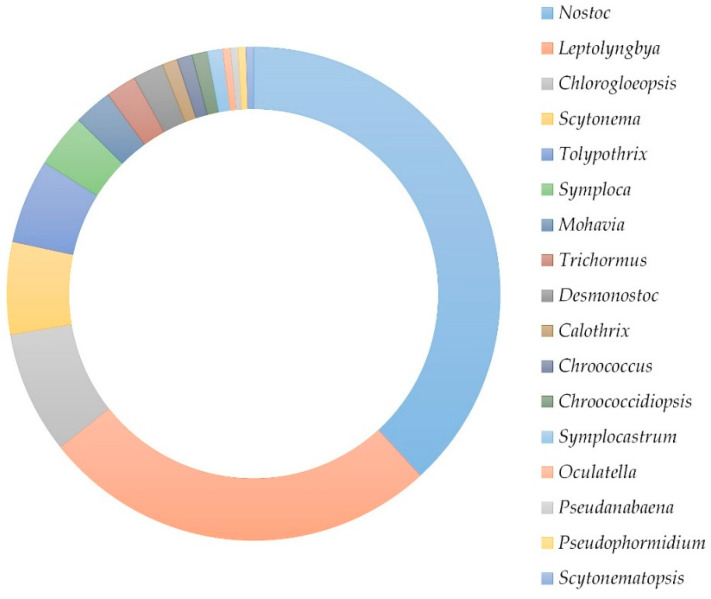
The distribution of identified genera. The most frequently isolated genera were *Nostoc* and *Leptolyngbya*, followed by *Chlorogloeopsis*, *Scytonema*, and *Tolypothrix*.

Cyanobacteria isolated from BLC samples consisted of either mixed-strain or monocyanobacterial cultures from the following genera: *Leptolyngbya*, *Nostoc*, cf. *Mojavia*, *Trichormus*, *Chlorogloeopsis*, *Calothrix*, *Tolypothrix*, *Symploca*, *Scytonema*, *Chroococcus*, *Chroococcidiopsis*, *Desmonostoc*, *Symplocastrum*, *Oculatella*, *Pseudanabaena*, and *Pseudophormidium, Scytonematopsis*. The underlined genera involve previously described cyanotoxin producing strains. The highest number of isolated strains was determined for the genera *Nostoc* and *Leptolyngbya* (Figure 1). The distribution of genera within the cultures is shown in Supplementary Materials File S1.

### 2.1. Chromatographic Analyses

We could not detect any of the eight MCs, CYN, and BMAA in analyzed BLCs. Similarly, we could not detect any of the eight MCs, CYN, STX, and GTX2/3 in mixed-strain cyanobacterial cultures derived from BLCs. Chromatograms and fragmentation spectra/MRM chromatograms for all groups of reference material are provided in Supplementary Materials File S2.

### 2.2. PCR Analyses

**Figure 2:**
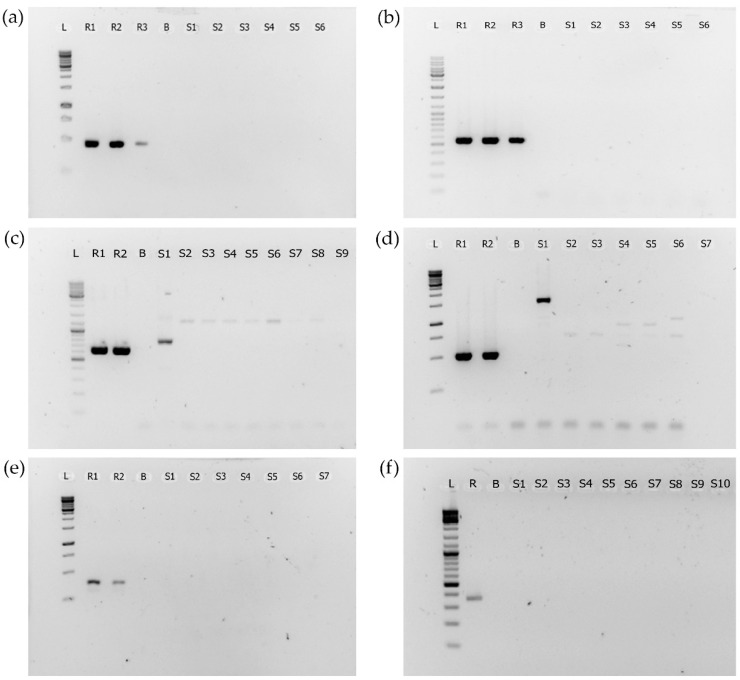
PCR amplification of target genes in reference material and lack of amplification in a number of samples. Legend: L—ladder; R—reference; B—blank; S—sample; (**a**) PCR amplification of mcyE/nodF genes in reference material. Primers used: HEPF and HEPR; reference material: R1: NIES-107 (*Microcystis*), R2: PCC7820 (*Microcystis*), and R3: PCC7806 (*Microcystis*); (**b**) PCR amplification of the *cyrJ* gene in reference material. Primers used: cyrJ_F and cyrJ_R; reference material: R1: CS-505 (*Cylindrospermopsis*), R2: CS-506 (*Cylindrospermopsis*), and R3: *Anabaena* 966; (**c**) PCR amplification of *sxtA* gene in reference material. Primers used: stxA855_F and sxtA1480_R; reference material: R1: CS-337/01 (*Dolichospermum*) and R2: CS-537/13 (*Dolichospermum*); (**d**) PCR amplification of sxtG gene in reference material. Primers used: sxtG432_F and sxtG928_R; reference material: R1: CS-337/01 (*Dolichospermum*) and R2: CS-537/13 (*Dolichospermum*); (**e**) PCR amplification of *sxtS* gene in reference material. Primers used: sxtS205_F and sxtS566_R; reference material: R1: CS-337/01 (*Dolichospermum*) and R2: CS-537/13 (*Dolichospermum*); (**f**) PCR amplification of *anaC* gene in reference material. Primers used: anaC-genF adn anaC-genR; reference material: *Anabaena* 123.

PCR amplification of the 16S RNA genes was successful in 55 out of 60 cyanobacterial strains. We could not detect PCR amplification of the genes coding the synthesis of MCs (*mcyE*), NOD (*nodF*), CYN (*cyrJ*), STX (*sxtA*, *sxtG*, and *sxtS*), and ANTX (*anaC*) in any of the 60 cyanobacterial strains isolated from BLCs. Images showing amplification of targeted genes in reference material are presented in Figure 2 (the raw gel images are provided in Supplementary Material S3).

## 3. Discussion

During this study, standard analytical and molecular biological methods developed for cyanobacterial samples from the aquatic environment were used. It is unlikely that the type of matrix (biomass and BLCs) affected the extraction and analysis of cyanotoxins and DNA, e.g., the extraction and instrumental analyses were based on widely-accepted protocols [32,33,34]. Interestingly, we could not detect any cyanotoxins, nor any cyanotoxin synthetase encoding genes in our study. This noteworthy contrast in toxin production between cyanobacteria inhabiting a specific terrestrial substrate, loess sediment, compared to aquatic environments is very intriguing and opens many questions about cyanotoxin production in cyanobacteria.

As there are cyanobacterial strains producing toxins and toxin-deficient strains, most cyanobacterial toxins have been regarded as secondary metabolites, i.e., compounds lacking essential functions in the growth of the organism under optimal conditions. Such compounds may still confer a certain selective advantage on the organism producing them and promote the long-term survival of the population. General information on the cause of toxin production in cyanobacteria and their functional biological/ecological role is still very limited and requires further research. However, several hypotheses have emerged over the years, supported by the results of various studies. Some possible biochemical and ecophysiological functions of cyanotoxins are shown in Table 1.

The fact that genes encoding the enzymes that produce MCs [66] and STX [67] appeared long before the appearance of algae, higher plants, and animal life on Earth implies that selection pressures other than predators/grazers and algal/plant competitors were likely responsible for their appearance. Different environmental conditions favor ecological/survival strategies and specific metabolic patterns with the highest adaptive value for these conditions. During their short activity phase, which depends on water availability, cyanobacteria in biocrusts of arid and semi-arid regions synthesize molecules for rapid recovery from desiccation and UV radiation damage, dial-regulated metabolism, and adaptation to next desiccation/resuscitation events [68], of which secondary metabolites are a significant part [69]. It may be that common cyanotoxins do not offer adaptive advantages under environmental conditions in which our BLC cyanobacteria occur.

Autoregulation and other biological phenomena have also been recognized as an ecological/survival strategy. There is increasing evidence of the release of microcystin as an essential part of the life cycle strategy of populations of *Microcystis* and other bloom-forming cyanobacteria [58,65,70,71]. Hu and Rzymski [70] examined apoptosis, i.e., programmed cell death, as an ecological/survival strategy of *Microcystis* and provided a conceptual model coupling apoptosis and MC release. Furthermore, the role of MCs and other non-ribosomal peptides in cell-to-cell communication during lytic events has been recognized. Schatz et al. [58] showed that lysis of *Microcystis* cells or exposure to the cyanobacterial metabolites MC, micropeptin, or microginin induced an enhanced production of MCs in the remaining *Microcystis* population. The authors interpreted this elevation of MC production and increased toxicity as an attempt to raise the fitness of the population in its ecological niche. MC involvement in the regulation of *Microcystis* morphotype (with influence on cell-to-cell contacts) was indicated by Zilliges et al. [65] who showed that the expression pattern of the extracellular glycoprotein *MrpC* was different in microcystin-containing and microcystin-deficient strains. Makower et al. [71] showed that the relative expression of genes related to the central intermediary metabolism, photosynthesis, and energy metabolism were different in a wild-type toxin-producing strain of *Microcystis aeruginosa* and a microcystin-deficient mutant. Interestingly, Makower et al. [71] showed that the signaling effect of extracellular, added microcystin was limited to the regulation of genes related to secondary metabolism only.

Successful cell-to-cell and especially colony-to-colony communication require a medium for the diffusion of autoinducers and other signaling molecules. It is evident that such communication cannot be equally successful or meaningful in areas with strongly limited water availability. Considering communication as an important biological function of MCs, it would be a waste of resources and an adaptive disadvantage for the cell to produce MCs in an environment where they cannot be utilized.

Could water availability actually explain the difference between our results and those of studies reporting cyanotoxins in biocrust cyanobacteria? Water availability varies widely in arid and semiarid regions. The BLCs and isolated cyanobacteria we studied originate predominantly from exposed vertical loess deposit profiles and hillslopes where water retention is very unlikely. However, some biocrusts develop in depressions where intense dew formation occurs or where rainwater can accumulate for some time. Assuming that sufficient water is available for a period of time, cyanobacteria would have a medium through which cyanotoxin-mediated signaling would be possible.

On the other hand, research on cyanotoxin production in biocrust cyanobacteria is insufficient, and we do not have a clear picture of the extent of cyanotoxin production in environments where biocrusts are present. It could simply be that the cyanobacterial strains studied here do not produce common cyanotoxins, as some cyanobacterial strains/species do in aquatic environments. As highlighted by Gärtner et al. [72] and Huang et al. [7], research on cyanotoxins in terrestrial environments should be more precise in terms of sampling site characteristics and conditions and species diversity if we are to fully understand the factors driving cyanotoxin production in terrestrial environments and the biochemical/ecophysiological role of cyanotoxins in general.

We assessed common cyanotoxins in strains of BLC cyanobacteria to evaluate their potential in restoration of degraded loess environments. Our results suggest that studied BLC cyanobacteria do not produce common cyanotoxins and imply their safe use in restoration. However, with recent developments in analytical techniques and in vitro and in vivo bioassays, bioactive compounds are being discovered that have toxic effects on human and animal cells but are structurally different from the known cyanotoxins, so we should not rule out the presence of some other toxic compounds [1,73]. For example, strains isolated from terrestrial environments have recently been reported to produce an uncommon cyanotoxin apratoxin, the production of which was previously associated exclusively with marine environments [7]. Bioassays could be a great tool to examine the general toxicity of inoculants [27,73], which is especially important for the rehabilitation of degraded and desertified land by cyanobacterial inoculation, since the application of toxic cyanobacterial strains would be hazardous.

One of the objectives of this study was to examine the biosignature capacity of cyanotoxins for use in paleoenvironmental studies of loess, in terms of genus/species specificity and abundance. Cyanotoxins are organism-specific compounds, and MCs and CYN have been reported to maintain stable structure under a variety of environmental conditions [35,74,75,76], which is a prerequisite for sedimentary biosignature. Cyanotoxins MCs and CYN have been used as a paleolimnological tool to detect past harmful algal blooms (HABs), track population fluctuations of cyanotoxin producers, and understand under which environmental conditions cyanotoxin production was triggered [77,78,79,80,81,82]. Although there are some concerns about the biomarker potential of MCs regarding stability (e.g., resistance of MCs to bacterial degradation under anoxic conditions) and extraction methods, they are considered to have great potential for use in paleolimnological studies along with CYN [83].

The notable absence of cyanotoxin production by the BLC cyanobacteria studied here suggests that cyanotoxin production is not as common in BLC and therefore may not be reliable biosignature candidates to represent terrestrial cyanobacterial populations in loess research. Further research is needed to understand the extent of cyanotoxin production in BLCs and terrestrial environments in general, and to link a particular terrestrial environment (e.g., loess) or environmental conditions to cyanotoxin production.

## 4. Conclusions

We did not detect targeted common cyanotoxins or cyanotoxin-encoding genes in the studied BLCs and cyanobacterial strains. This is an indication of either lack of common cyanotoxin production in studied BLC cyanobacteria or production of novel cyanotoxins/secondary metabolites. The environment of biocrust cyanobacteria is completely different from that of aquatic cyanobacteria. Therefore, different metabolic profiles might be expected, as metabolites may not have the same adaptive value in different environments. To date, little research has been conducted on the production of cyanotoxins by terrestrial cyanobacteria, and we hope that this article will stimulate further studies on this topic. This is of great importance if we are to define the biochemical or ecophysiological roles of cyanotoxins.

Furthermore, our results suggest that analyzed cyanobacterial strains can potentially be used in land restoration. We suggest that cyanobacterial strains should be tested with e.g., bioassays prior to inoculation in the field to investigate their potential toxicity, as they may produce non-common toxic compounds or toxic compounds not yet described.

The observed absence of cyanotoxin production in studied BLC cyanobacteria does not favor the use of cyanotoxins as a biosignature of cyanobacterial communities in the reconstruction of paleoclimate and paleoenvironment in loess. Further studies are needed to link specific environments to cyanotoxin occurrence in terrestrial sediment records.

## 5. Material and Methods

### 5.1. Biocrust Samples and Cyanobacterial Cultures

The biocrust samples and cyanobacterial cultures with strain identifications to the genus level are presented in Supplementary Materials File S1. Biocrusts were sampled from the exposed loess beds and exposed vertical loess profiles in Serbia 2006 (Ruma, old brickyard; Titel Loess Plateau (TLP)—Mošorin); exposed vertical loess profiles in Serbia 2015 (Ruma, old brickyard; Irig; TLP—Vilovo; TLP—Titel old brickyard; Stari Slankamen); exposed vertical loess profiles in China 2013 (Luochuan; Xifeng; Zhaojiachuan); and exposed vertical loess profiles and upland loess slopes in Iran 2014 (Saravan; Neka; Toshan; Gorgan; Now Deh; Agh Band). Samples were stored in dry and dark conditions at room temperature (≅22 °C) until cyanotoxin analyzes in 2015 and 2016. The size of biocrust samples varied between 5 cm^2^ and 15 cm^2^.

Prior to isolation of cyanobacterial strains, biocrust samples were visually examined using binocular stereomicroscope (Leica MZ) to confirm the presence of cyanobacterial colonies. To isolate cyanobacteria, approximately two cm^2^ of a BLC was soaked in 50 mL of BG11-N and BG11-N_0_ (nitrate-free) medium [84] and cultured under a 14/10 (light/dark) illumination period (8000 K, 60 lux, 750 lm; Power-GLO, Rolf C. Hagen Inc., Mansfield, MA, USA) at room temperature (≅22 °C) for 30 days. During this period, colonies of cyanobacteria formed over and around the biocrust sample. The colonies were separated from the biocrust surface and flask walls/bottom and transferred to 5 mL of fresh BG11-N/N_0_. After 30 days of cultivation under the conditions mentioned above, 40 mL of fresh BG11-N/N_0_ medium was added to the culture. After 30 days of cultivation, 30 mL of culture medium was removed, and another 30 mL of BG11-N/N_0_ medium was added to the culture. The substitution of the culture media was repeated monthly until further analysis. A total of 98 mixed-strain cultures were established.

In further steps, a total of 60 cyanobacterial strains were isolated to form the monocyanobacterial cultures. Isolation and purification were conducted through a series of alternate cultivation in liquid and solid (1.5% agar) BG-N₀ media [85]. Finally, the isolated strains were cultured in 50 mL of BG-N₀ medium in an incubator under a 14/10 (light/dark) illumination period (8000 K, 60 lux, 750 lm; Power-GLO, Rolf C. Hagen Inc., Mansfield, MA, USA) at 27 °C during four weeks. Approximately 10 mL of each strain culture was freeze-dried before extraction. 

The identification of cyanobacteria to the genus level was performed on VWR BI 100 (VWR International, Belgium), Olympus BX 50, and Olympus BX 51 microscopes (400–1000× magnification) using identification keys [86,87,88].

### 5.2. Extraction of Cyanotoxins

Extraction of microcystins was performed on ten biocrust samples collected in Serbia and 98 mixed-strain cultures. Extraction of cylindrospermopsin was performed on ten biocrust samples collected in Serbia and 35 mixed-strain cultures. Extractions were performed according to SOP_TOXIC_AAU_04F [89] with minor modifications. For the extraction of cyanotoxins from the biocrusts, approximately 1 g of the biocrust surface from paired samples containing cyanobacteria was scraped and soaked with 5 mL of 75% MeOH (J.T. Baker HPLC Gradient Grade, The Netherlands). Extraction continued with 15 min sonication in an ultrasonic bath, followed by 1 min sonication with a probe (3 mm microtip, 30% pulse, 30% energy; Bandelin Sonopuls HD2070). To avoid contamination, the probe was washed with 75% MeOH after each sample. Samples were then placed in a water bath at 50 °C for 30 min with shaking. The centrifugation at 2000× *g* for 15 min followed, and 3 mL of the supernatant was separated and evaporated to dryness under a stream of nitrogen at 50 °C. For analysis of cylindrospermopsin from the same vial, the extracts were dissolved with 300 µL of 50% MeOH (LC-MS Chromasolv, Riedel-de Haën™, Germany) and vortex shaken for 30 s. Finally, the extracts were filtered through a GHP ACRODISC ⌀13 mm syringe filter with a 0.2 µm GHP membrane (Pall Corporation, New York, NY, USA). 

For extraction of the same cyanotoxins from cyanobacteria in mixed cultures, approximately 100 mL of the culture was filtered through Whatman GF/C ⌀47 mm glass microfiber filters (GE Healthcare UK Limited, Hatfield, UK). The biomass retained on the filters (50.2–141 mg) was freeze-dried and then dissolved in 3 mL of 75% MeOH (J.T. Baker HPLC Gradient Grade, Phillipsburg, NJ, USA). Samples were further sonicated for 15 min in an ultrasonic bath, followed by 1 min sonication with a probe (3 mm microtip, 30% pulse, 30% energy; Bandelin Sonopuls HD2070, Sigma-Aldrich, Burlington, MA, USA). The probe was washed with 75% MeOH after each sample. A volume of 1.5 mL from each sample was transferred to Eppendorf tubes and centrifuged at 10,000× *g* for 10 min. A volume of 1 mL of the supernatant was evaporated to dryness under a nitrogen stream at 50 °C. Samples were further processed in the same manner as biocrust samples.

Extraction of saxitoxin and gonyautoxin 2-, and 3 hydrochloride from 10 mixed-strain cultures was performed in 80% acetonitrile (ACN) containing 0.1% formic acid. First, the samples were sonicated in a bath for 15 min, after which each sample was sonicated separately with a probe (3 mm microtip, 30% pulse, 30% energy; Bandelin Sonopuls HD2070). Samples were then centrifuged at 10,000× *g* for 10 min. The supernatant was separated and filtered through a GHP Acrodisc Ø13 mm syringe filter with a 0.2 µm GHP membrane. The filtered extracts were stored at −20 °C until two layers were separated (upper-ACN; lower-H_2_O). The lower layer was separated and submitted to HPLC analysis.

BMAA extraction was based on [13]. First, 600 µL of 0.1 M trichloroacetic acid (TCA) was added to 12 mg of cyanobacterial BLC sample. After vortexing and standing at room temperature for 10 min, the sample was vortexed and centrifuged. The supernatant was transferred to a spin filter tube and centrifuged again for 5 min. The filtrate was transferred to a plastic tube. Then, 600 µL of TCA was again added to the pellet of the sample, and the extraction and centrifugation steps were repeated. The pooled filtrate was split for the extraction of free and soluble bound BMAA. For free BMAA extraction, 20 µL of a 2 mg/L D_3_BMAA solution in 20 mM HCl was added to 500 µL extract. These samples were dried under vacuum, and then reconstituted in 500 µL 67% acetonitrile/33% water/0.1% formic acid and transferred to a vial for analysis. For soluble bound BMAA, 450 µL of the pooled filtrate was transferred to a small glass vial. Next, 20 µL of D_3_BMAA solution was added and the samples were dried under vacuum. Then, 30 µL 6 M HCl was added, after which the samples were flushed with nitrogen and hydrolyzed at 105 °C for 20 h. After hydrolysis, the samples were again dried under vacuum and reconstituted in two times 250 µL 67% acetonitrile/33% water/0.1% formic acid. After centrifugation in a spin filter tube, the samples were transferred to a vial for analysis. Total BMAA was extracted by adding 20 µL D_3_BMAA solution to 0.5 mg of sample. The samples were dried, and then hydrolyzed in the same way as the extracts for soluble bound BMAA.

### 5.3. Chromatographic Analyses

Liquid chromatography-tandem mass spectrometry (LC-MS/MS) was performed to analyze the presence of eight MCs (MC-LR, dmMC-LR, MC-RR, dmMC-RR, MC-LY, MC-LW, MC-YR, and MC-LF) and CYN in the samples. The analyses of MCs in 98 mixed-strain cultures and 20 BLCs sampled in Serbia were carried out according to [90,91] on an Agilent 1200 Rapid Resolution (RR) LC coupled to ion trap mass spectrometer with electrospray ion (ESI) source (Bruker Daltonics HCT Ultra, Bremen, Germany). Extracts were loaded (5 µL injection volume) onto Ascentis C_18_, 50 mm × 3 mm I.D. column with 3 µm particles (Supelco) at 40 °C. Solvent A was H_2_O-ACN-formic acid (99:1:0.1, *v*/*v*/*v*) and Solvent B was ACN-formic acid (100:0.1, *v*/*v*). The following linear solvent gradient program was used: 0 min 25% B, 5 min 70% B, 6 min 70% B, and 6.1 min 25% B; stop time 10 min with flow rate 0.5 mL/min. Data acquisition was performed using Compass 1.3 software (Bruker Daltonics, Bremen, Germany). The mass spectrometer (MS) was operating under following conditions: positive electrospray ion trap mode; dry temperature 350 °C; dry gas 10.0 l/min; nebulizer pressure 40 psi; capillary voltage 4.0 kV; scan range from *m*/*z* 500 to *m*/*z* 1200 with the Smart parameter Setting (SPS) function; the ICC target 300,000 with a maximum accumulation time of 100 ms; MS–MS fragmentation assisted by Smart Frag setting. The analyzes of CYN in 35 mixed-strain cultures and 11 BLCs sampled in Serbia were carried out with small modifications to the LC and MS methods used in the analyzes of MCs. The linear solvent gradient program was 0 min 100% A, 2.5 min 100% A, 2.6 min 50% A, 4 min 50% A, 4.1 min 100% A; stop time 10 min; flow rate 0.5 mL/min. The MS scan ranged from *m*/*z* 395 to *m*/*z* 440 with the Smart parameter Setting (SPS) function. The ICC target was set to 200,000 with a maximum accumulation time of 100 ms. 

The analyses of GTX 2/3 and STX in ten mixed-strain cultures were carried out using the ion-pair HPLC with post-column oxidation and fluorescence detection (HPLC-FLD) according to [92] on a Merck Hitachi LaChrom HPLC-system (Tokyo, Japan) coupled to a Hewlett-Packard Series 1100 Fluorescence detector. Extracts were loaded (10 µL injection volume) onto Waters Xbridge C18 150 mm × 3 mm I.D. column with 3.5 µm particles (Milford, MA, USA) at 40 °C. Solvent A was (6 mM octanesulfonic acid, 6 mM heptanesulfonic acid and 40 mM ammonium phosphate, 20% phosphoric acid, pH 7)-tetrahydrofuran, 99.25:0.75 (*v*/*v*). Solvent B was (7 mM octanesulfonic acid, 7 mM heptane sulfonic acid, 48 mM ammonium phosphate, 20% phosphoric acid, pH 7)-tetrahydrofuran-acetonitrile, 89:1:10 (*v*/*v*/*v*). The following pump gradient program was used: 0 min 100% A, 6 min 100% A (GTX2/3 elute), 7.5 min 100% B, 32 min 100% B (STX elue), 33 min 100% A, 45 min 100% A, and flow rate 0.55 mL/min. The oxidized derivatives of STX and GTX2/3 were detected at *λ*_Ex_ 330 nm and *λ*_Em_ 395 nm, following the post-column oxidation: 60 °C in a PTFE reaction coil (15 m × 0.3 mm I.D.) with 5 mM periodic acid and 275 mM ammonia in water at 0.3 mL/min. 0.38 M nitric acid at 0.4 mL min^−1^, was used to lower the pH to acidic.

In-house prepared microcystin reference materials containing MC-LR, dmMC-LR, MC-RR, dmMC-RR, MC-LY, MC-LW, MC-YR, and MC-LF obtained from PCC7820 *Microcystis* and NIES-107 *Microcystis* strains were used for identification of MCs [93]. Certified reference materials for CYN, STX and gonyautoxin 2-, and 3 hydrochloride (GTX2/3) were acquired from NRC-IMB (Institute for Marine Biosciences, Halifax, Canada). Table 2 shows the lowest analyte levels in the diluted reference materials which gave a signal-to-noise ratio (S/N) higher than three.

BMAA analysis was performed as in [13]. Samples were analyzed without derivatization by LC-MS/MS. Quantification was performed against a calibration curve in solvent, and each sample was corrected for the recovery of the internal standard D_3_BMAA. Table 3 shows limit of detection (LOD) and limit of quantification (LOQ) values for BMAA.

### 5.4. DNA Extraction

Screening for cyanotoxin-coding genes was performed on 60 monocyanobacterial cultures (20:20:20 Serbia:Iran:China, Supplementary Materials File S1). Approximately 10 mg of freeze-dried biomass of both reference strains and culture samples was used for DNA extraction. Genomic DNA was extracted using the DNeasy Plant Mini Kit (QIAGEN, Hilden, Germany) according to the manufacturer’s instructions. The quality and quantity of the extracts were assessed spectrophotometrically (NanoDrop ND-1000, Thermo Scientific, Waltham, MA, USA) through the A_260_/A_280_ ratio.

### 5.5. Polymerase Chain Reaction (PCR)

Qualitative PCR was run to analyze samples for the presence of MC (*mcyE*), CYN (*cyrJ*), STX (*sxtA*, *sxtG*, and *sxtS*), and ANTX (*anaC*) synthetase genes in 60 cyanobacterial strains. The presence of cyanobacterial DNA in the samples was confirmed by PCR amplification of 16S rRNA gene. PCR reaction mixtures were prepared in a total volume of 20 µL containing 1 × Phire Reaction Buffer, 0.4 µL Phire II HotStart polymerase (Thermo Scientific, Waltham, MA, USA), 0.2 mM dNTPs (Thermo Scientific), 0.5 µL forward and reverse primers (Table 4), 2 µL of the template containing 0.35 to 0.5 ng/µL of genomic DNA, and sterile deionized water. The PCR protocol for the amplification of the 16S RNA gene [94] included: initial denaturation for 5 min at 94 °C; 30 cycles of denaturation for 45 s at 94 °C, primer annealing for 45 s at 57 °C, and strand elongation for 2 min at 68 °C; final elongation for 7 min at 68 °C. The PCR protocol for genes coding cyanotoxin production included: initial denaturation for 30 s at 98 °C; 40 cycles of denaturation for 5 s at 98 °C, primer annealing for 5 s at 52 °C or 61 °C or 62 °C (Table 4), strand elongation for 10 s at 72 °C; final elongation of 1 min at 72 °C (C1000 Touch Thermal Cycler (Bio-Rad, Hercules, CA, USA)). Potential inhibition of PCRs was assessed through an exogenous amplification control template containing 1 µL: 1 µL (reference: sample). Following strains were used as a reference in the control template: PCC7820 for *mcyE*, CS-506 for *cyrJ*, CS-537/13 for *sxtA*, *sxtG*, *sxtS*, and ANA123 for *anaC*. Visualization of PCR products was performed on a 1.5% Top Vision agarose gel (Thermo Scientific) dyed with SYBR^®^ Safe DNA gel stain. The bands were documented on Gel Doc™ XR (Bio-Rad, Hercules, CA, USA) using Quantity One software (v. 4.6.9) (Bio-Rad, Hercules, CA, USA). 

### 5.6. Reference Strains for PCR Analysis

The reference strains for PCR analysis were obtained from Finnish Environment Institute (SYKE), Pasteur Culture Collection (PCC), Australian National Algae Culture Collection (CS), University of Helsinki Culture Collection (UHCC), and National Institute for Environmental Studies Microbial Culture Collection (NIES). MC reference strains: NIES-107 (*Microcystis*), PCC7820 (*Microcystis*), and PCC 7806 (*Microcystis*); CYN reference strains: CS-505, CS-506 (*Cylindrospermopsis*), and *Anabaena* 966 (SYKE); SXT reference strains: CS-337/01 and CS-537/13 (*Dolichospermum*); ANA-a reference strain: *Anabaena* 123 (UHCC) were used in this study.

## Figures and Tables

**Table 1:** Possible biochemical and ecophysiological roles of cyanotoxins that have been reported in the literature.

	Possible Biological Functions	References
Competitive advantage	Defense mechanism against predators/grazers	[35,36,37,38,39,40,41,42]
	Competitive interactions with microalgae	[43,44,45,46]
	Competitive interactions with cyanobacteria	[43,46,47,48]
	Competitive interactions with aquatic and terrestrial plants	[49,50,51,52,53]
Cellular physiology benefits	Tools in the acquisition and retention of nutrients	[44,54,55,56,57,58]
	Attractants/repellents for heterotrophic microorganisms	[59]
	Stress response (infochemicals and radical scavengers)	[58,60,61,62,63,64,65]

**Table 2:** Detectable cyanotoxin levels in the reference materials and corresponding toxin levels in dry cyanobacterial material.

Toxin	Lowest Cyanotoxin Levels in the Diluted Reference Materials Giving S/N > 3 (pg/µL) *	Corresponding Toxin Levels in Dry Cyanobacterial Material (µg/g)
dmMC-RR	3.8	0.030
MC-RR	11.2	0.089
MC-YR	12.4	0.097
dmMC-LR	10.0	0.079
MC-LR	10.7	0.085
MC-LY	11.1	0.088
MC-LW	34.6	0.27
MC-LF	22.0	0.18
CYN	125	0.98
STX	2.4	0.002
GTX2/3 **	453/171	0.36/0.13

* these values do not represent limits of detection but detectable concentrations present in the 100–1000× diluted reference materials, and ** standard dilutions of more than 100× were not run for the analysis of GTX2/3.

**Table 3:** Detection and quantification limits for BMAA.

	Free	SB	Tot
LOD (µg/g dw)	0.2	0.2	2
LOQ (µg/g dw)	0.5	0.6	5

**Table 4:** List of primers used for qualitative PCR.

Gene	Primer	5′-3′ Sequence	Annealing t (°C)	Reference
16S RNA	pA 23S30R	AGAGTTTGATCCTGGCTCAG CTTCGCCTCTGTGTGCCTAGGT	57	[95,96]
mcyE	HEPF HEPR	TTTGGGGTTAACTTTTTTGGGCATAGTC AATTCTTGAGGCTGTAAATCGGGTTT	61	[97]
cyrJ	cyrJ_F cyrJ_R	TTCTCTCCTTTCCCTATCTCTTTATC GCTACGGTGCTGTACCAAGGGGC	62	[98]
sxtA	stxA855_F sxtA1480_R	GACTCGGCTTGTTGCTTCCCC GCCAAACTCGCAACAGGAGAAGG	61	[92]
sxtG	sxtG432_F sxtG928_R	AATGGCAGATCGCAACCGCTAT ACATTCAACCCTGCCCATTCACT	62	[92]
sxtS	sxtS205_F sxtS566_R	GGAGTATTDGCGGGTGACTATGA GGTGGCTACTTGGTATAACTCGCA	62	[99]
anaC	anaC-genF anaC-genR	TCTGGTATTCAGTCCCCTCTAT CCCAATAGCCTGTCATCAA	52	[100]

## Data Availability

The data presented in this study are available on request from the corresponding author T.D. Some of the data are not publicly available due to later utilization in PhD thesis.

## Supplementary Materials

The following supporting information can be downloaded at: https://www.mdpi.com/article/10.3390/toxins14030215/s1, Supplementary Materials File S1: Mixed-strain cultures of cyanobacteria isolated from BLC; Supplementary Materials File S2: Chromatograms and fragmentation spectra/MRM chromatograms for all groups of reference material; Supplementary Materials File S3: Raw gel images of PCR amplification of target genes in reference material and lack of amplification in a number of samples.
