# Supplementary material for: Assessment of Common Cyanotoxins in Cyanobacteria of Biological Loess Crusts
Source: Toxins (Basel). 2022 Mar 16;14(3):215. doi: 10.3390/toxins14030215 (PMC8953721; doi:10.3390/toxins14030215)
Supplement: Supplementary file 1 [file toxins-14-00215-s001.zip › Supplementary Material S1.pdf]

Supplementary Material S1. Mixed-strain cultures of cyanobacteria isolated from BLC.

Table S1. Mixed-strain cultures of cyanobacteria isolated from BLC samples from China. Genera in the table are the predominant genera in the mixed culture (assessment based on visual inspection); genera in bold denote the strains used in the PCR analyses. CTs: cyanotoxins analysed in each culture.

China										
**SAMPLE NAME**	**GP1**	**GP2**	**GP3**	**GP4**	**GP5**	**GP6**	**GP7**	**GP8**	**GP9**	**GP10**
**GENERA**	*Nostoc*<br>*Nostoc*	***Nostoc***	***Nostoc***	*Nostoc*<br>*Leptolyngbya*	*Leptolyngbya*<br>*Chlorogloeopsis*	*Leptolyngbya*<br>***Nostoc***<br>*Chlorogloeopsis*	***Leptolyngbya***<br>*Calothrix*	*Leptolyngbya*<br>*Nostoc*	*Leptolyngbya*<br>*Tolypothrix*<br>*Trichormus*	**cf. *Mojavia***
**CTs**	MCs	MCs	MCs	MCs	MCs	MCs	MCs	MCs	MCs	MCs
**SAMPLE NAME**	**GP11**	**GP12**	**GP13**	**GP14**	**GP15**	**GP16**	**GP17**	**GP18**	**GP19**	**GP20**
**GENERA**	***Nostoc***<br>*Leptolyngbya*	*Leptolyngbya*<br>*Nostoc*	*Nostoc*	*Nostoc*	*Nostoc*<br>*Leptolyngbya*	*Leptolyngbya*<br>*Nostoc*	*Leptolyngbya*<br>*Chlorogloeopsis*	*Leptolyngbya*	*Leptolyngbya*<br>***Chlorogloeopsis***	***Chlorogloeopsis***
**CTs**	MCs	MCs	MCs	MCs	MCs	MCs	MCs	MCs	MCs	MCs

SAMPLE NAME	LR1	LR2	LR3	LR4	LR5	LR6	LR7	LR8	LR9	LR10
GENERA	***Leptolyngbya***	*Leptolyngbya*<br>*Symploca*	n.d.	*Symploca*<br>*Leptolyngbya*	*Nostoc*<br>*Symploca*	*Nostoc*	*Symploca*<br>***Nostoc***	*Desmonostoc*	*Leptolyngbya*<br>*Nostoc*	***Chlorogloeopsis***<br>*Leptolyngbya*
CTs	MCs	MCs	MCs	MCs	MCs	MCs	MCs	MCs	MCs	MCs

*table continues

**SAMPLE NAME**	**LR11**	**LR12**	**LR13**	**LR14**	**MC1**	**MC2**	**MC3**	**MC4**	**MC5**	**MC6**
**GENERA**	*Nostoc*<br>*Leptolyngbya*<br>*Chroococcus*	*Leptolyngbya*<br>*Nostoc*	*Nostoc*<br>*Leptolyngbya*<br>*Chlorogloeopsis*	*Nostoc*<br>*Chlorogloeopsis*	*cf. Mojavia*	*Nostoc*<br>*Symploca*	***Leptolyngbya***	*Leptolyngbya*<br>***Nostoc***	*Nostoc*	*Nostoc*<br>*Leptolyngbya*<br>*Tolypothrix*
**CTs**	MCs	MCs	MCs	MCs	MCs<br>CYN	MCs<br>CYN	MCs<br>CYN	MCs<br>CYN	MCs<br>CYN	MCs<br>CYN

**SAMPLE NAME**	**MC7**	**MC8**	**MC9**	**MC10**	**MC11**	**MC12**	**LP1**	**LP2**	**LP3**	**LP4**
**GENERA**	*Nostoc*<br>*Leptolyngbya*<br>***Tolypothrix***	*Nostoc*<br>*Leptolyngbya*<br>*Tolypothrix*	*Nostoc*<br>*Leptolyngbya*<br>*Tolypothrix*	*Nostoc*<br>*Leptolyngbya*<br>*Tolypothrix*	***Nostoc***<br>*Leptolyngbya*<br>*Tolypothrix*	*Nostoc*<br>*Tolypothrix*	*Leptolyngbya*<br>*Nostoc*	***Nostoc***	*Nostoc*<br>*Trichormus*	*Trichormus*
**CTs**	MCs<br>CYN	MCs<br>CYN	MCs<br>CYN	MCs<br>CYN	MCs<br>CYN	MCs<br>CYN	MCs	MCs	MCs	MCs

**SAMPLE NAME**	**LP5**	**LP6**	**LP7**	**LP8**	**XFU**	**XFU1**	**XFU3**	**ZJ1**	**ZJ2**	**ZJ3**
**GENERA**	*cf. Mojavia*	*Leptolyngbya*<br>*Nostoc*<br>*cf. Mohavia*	*Leptolyngbya*<br>*Nostoc*<br>*cf. Mohavia*	***Nostoc***	*Nostoc*<br>*Scytonema*<br>*Calothrix*	*Leptolyngbya*<br>*Symploca*<br>*Nostoc*	***Tolypothrix***	***Leptolyngbya***<br>***Nostoc***	*Leptolyngbya*	*Scytonema*
**CTs**	MCs	MCs	MCs	MCs	MCs	MCs	MCs	MCs<br>CYN	MCs<br>CYN	MCs<br>CYN

**SAMPLE NAME**	**ZJ4**	**ZJ5**	**ZJ6**	**ZJ7**	**ZJ8**	**ZJ9**
**GENERA**	*Nostoc*<br>*Leptolyngbya*<br>*Scytonema*	*Leptolyngbya*	*Nostoc*	***Nostoc***	*Symploca*	***Nostoc***
**CTs**	MCs<br>CYN	MCs<br>CYN	MCs<br>CYN	MCs<br>CYN	MCs<br>CYN	MCs<br>CYN

Table S1.2. Mixed-strain cultures of cyanobacteria isolated from BLC samples from Iran. Genera in the table are the predominant genera in the mixed culture (assessment based on visual inspection); genera in bold denote the strains used in the PCR analyses. CTs: cyanotoxins analysed in each culture.

Iran

**SAMPLE NAME**	**IRN1A**	**IRN1B**	**IRN2**	**IRN3**	**IRN4**	**IRN5A**	**IRN5B**	**IRN5C**	**IRN5D**	**IRN5E**	**IRN5F**
**GENERA**	***Tolypothrix***	***Nostoc***<br>***Leptolyngba***	***Nostoc***	***Nostoc***	*Nostoc*<br>***Trichormus***	***Nostoc***<br>***Nostoc***	*Leptolyngbya*<br>*Nostoc*	***Leptolyngbya***<br>*Scytonema*	***Nostoc***	*Chlorogloeopsis*	*Chlorogloeop.*
**CTs**	MCs<br>CYN	MCs<br>CYN	MCs<br>CYN	MCs<br>CYN	MCs<br>CYN	MCs<br>CYN	MCs<br>CYN	MCs<br>CYN	MCs<br>CYN	MCs<br>CYN	MCs<br>CYN

**SAMPLE NAME**	**IRN5G**	**IRN5H**	**IRN5I**	**IRN5J**	**IRN5K**	**IRN6**	**IRN7**	**IRN8**	**IRN9A**	**IRN9B**
**GENERA**	***Desmonostoc***	*Nostoc*	***Desmonostoc***<br>***Nostoc***	*Leptolyngbya*<br>*Tolypothrix*<br>***Chlorogloeopsis***	***Desmonostoc***	*Leptolyngbya*<br>***Nostoc***<br>*Pseudophormidium*	*Leptolyngbya*<br>***Scytonema***	***Nostoc***	**Chlorogloeopsis**	***Nostoc***
**CTs**	MCs<br>CYN	MCs<br>CYN	MCs<br>CYN	MCs<br>CYN	MCs<br>CYN	MCs	MCs	MCs	MCs	

Table S1.3. Mixed-strain cultures of cyanobacteria isolated from BLC samples from Serbia. Genera in the table are the predominant genera in the mixed culture (assessment based on visual inspection); genera in bold denote the strains used in the PCR analyses. CTs: cyanotoxins analysed in each culture.

Serbia									
**SAMPLE NAME**	**SSS1**	**SSS2**	**SSS4**	**SSS5**	**R1**	**R3**	**R4**	**R5**	**R6**
**GENERA**	***Leptolyngbya***<br>*Nostoc*	*Nostoc*<br>*Leptolyngbya*	***Nostoc***	*Nostoc*<br>***Symplocastrum***	*Nostoc*<br>***Chlorogloeopsis***	*Nostoc*<br>*Chlorogloeopsis*	***Scytonema***<br>*Nostoc*	*Nostoc*<br>***Chlorogloeop.***	***Scytonematopsis***<br>*Symplocastrum*
**CTs**	MCs	MCs	MCs	MCs	MCs	MCs	MCs	MCs	MCs

**SAMPLE NAME**	**M1**	**M2**	**LES1**	**LES2**	**LES3**	**LES4**	**LES5**	**LES6**	**LES7**
**GENERA**	*Leptolyngbya*<br>***Nostoc***	*Nostoc*	***Chroococcidiopsis***	***Nostoc***	*Scytonema*<br>*Nostoc*	***Scytonema***<br>*Chroococcus*<br>***Nostoc***	***Nostoc***<br>***Scytonema***	***Scytonema***<br>***Nostoc***	***Leptolyngbya***<br>*Nostoc*
**CTs**	MCs	MCs	MCs<br>STX<br>GTX2/3	MCs<br>STX<br>GTX2/3	MCs<br>STX<br>GTX2/3	MCs<br>STX<br>GTX2/3	MCs<br>STX<br>GTX2/3	MCs<br>STX<br>GTX2/3	MCs<br>STX<br>GTX2/3

**SAMPLE NAME**	**LES8**	**V**	**SL**	**R2**	**LES5-1**	**TLP VIL**	**SSSu**	**RUMA**	**IRIG**

**GENERA**	*Nostoc*<br>*Leptolyngbya*	*Leptolyngbya*<br>*Oculatella*	*Nostoc*<br>*Pseudanabaena*	*Nostoc*<br>*Scytonema*	*Scytonema*	***Chroococcus***<br>*Nostoc*	***Leptolyngbya***<br>*Nostoc*	***Chlorogloeopsis***<br>*Leptolyngbya*	*Leptolyngbya*<br>*Nostoc*
**CTs**	MCs<br>STX<br>GTX2/3	MCs	MCs	STX<br>GTX2/3	STX<br>GTX2/3				
