# Supplementary material for: Assessment of Common Cyanotoxins in Cyanobacteria of Biological Loess Crusts
Source: Toxins (Basel). 2022 Mar 16;14(3):215. doi: 10.3390/toxins14030215 (PMC8953721; doi:10.3390/toxins14030215)

Supplementary Material S2

- Extracted ion chromatogram and fragmentation spectrum of MC-LR (m/z 995) in PCC7820
- Extracted ion chromatogram and fragmentation spectrum of CYN (m/z 416) (CRM)
- Chromatograms of STX and GTX2/3 (CRM)
- MRM Chromatogram of BMAA, DAB, AEG, and D3BMAA

Figure S1. Extracted ion chromatogram (a) and fragmentation spectrum (b) of MC-LR in PCC7820

a)

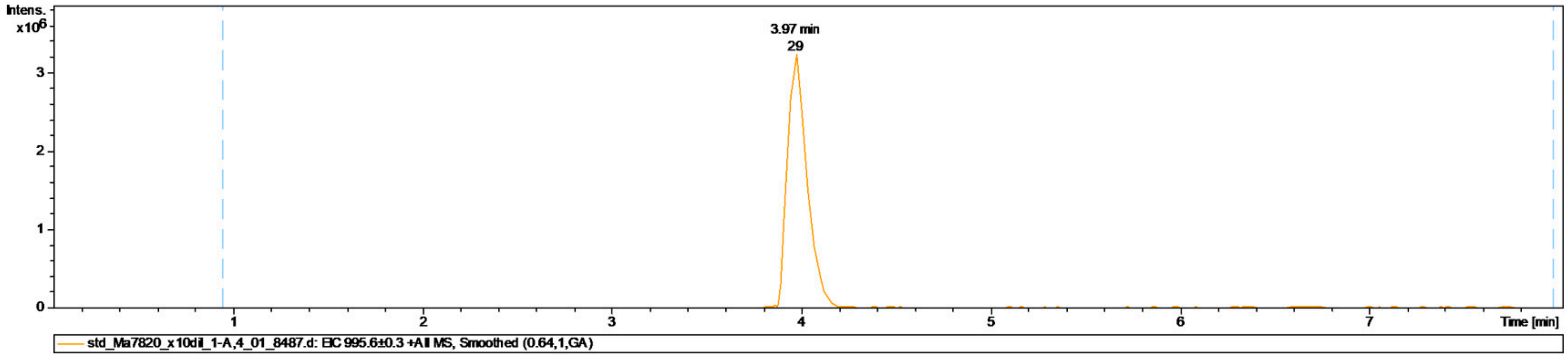

b)

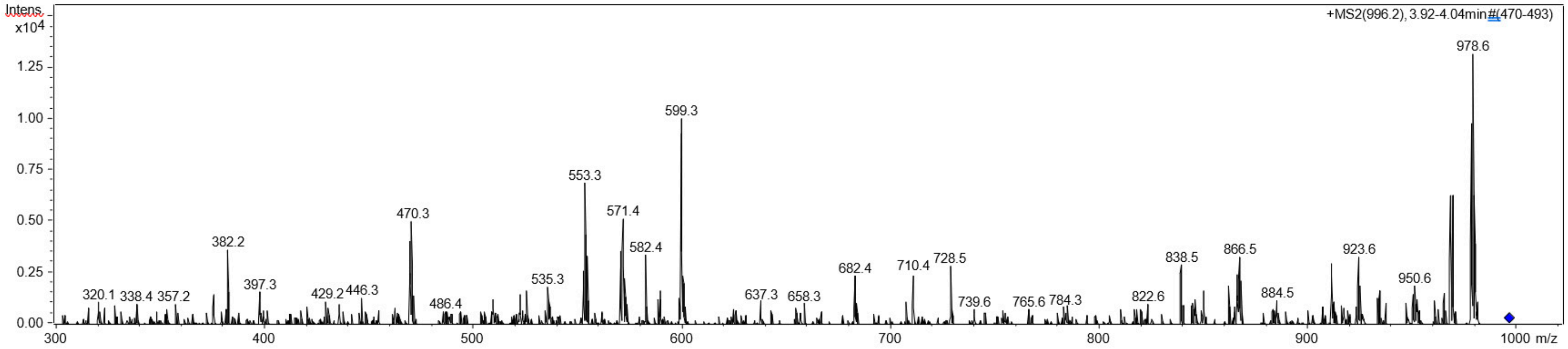

Figure S2. Extracted ion chromatogram (a) and fragmentation spectrum (b) of Cylindrospermopsin (CRM) reference

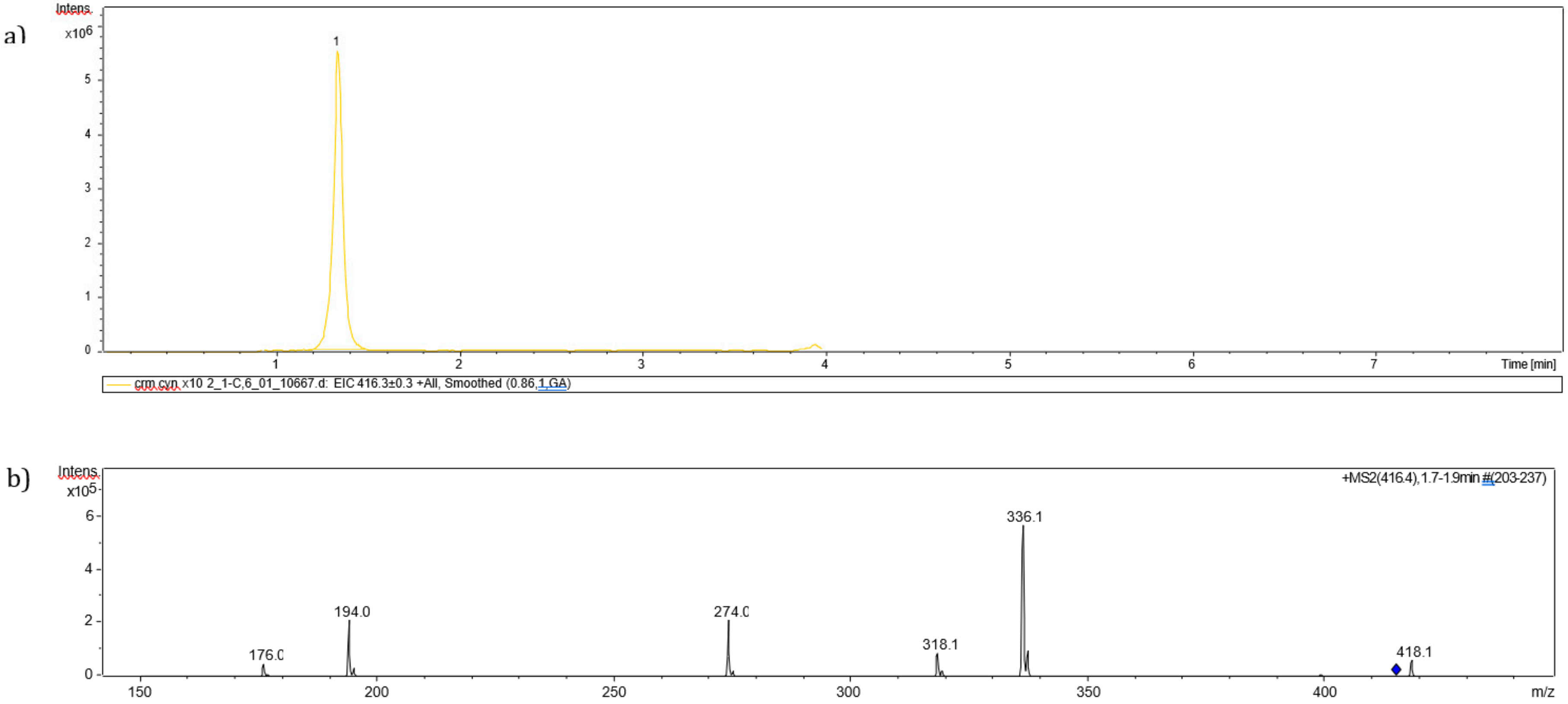

Figure S3. Chromatogram of STX (CRM)

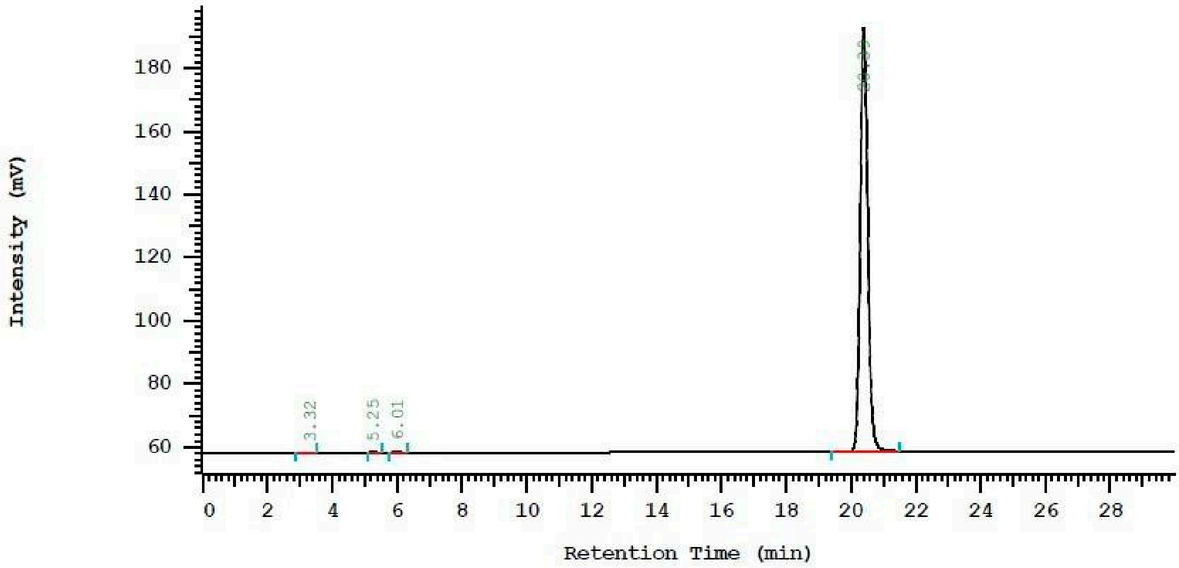

Figure S4. Chromatogram of GTX2/3 (CRM)

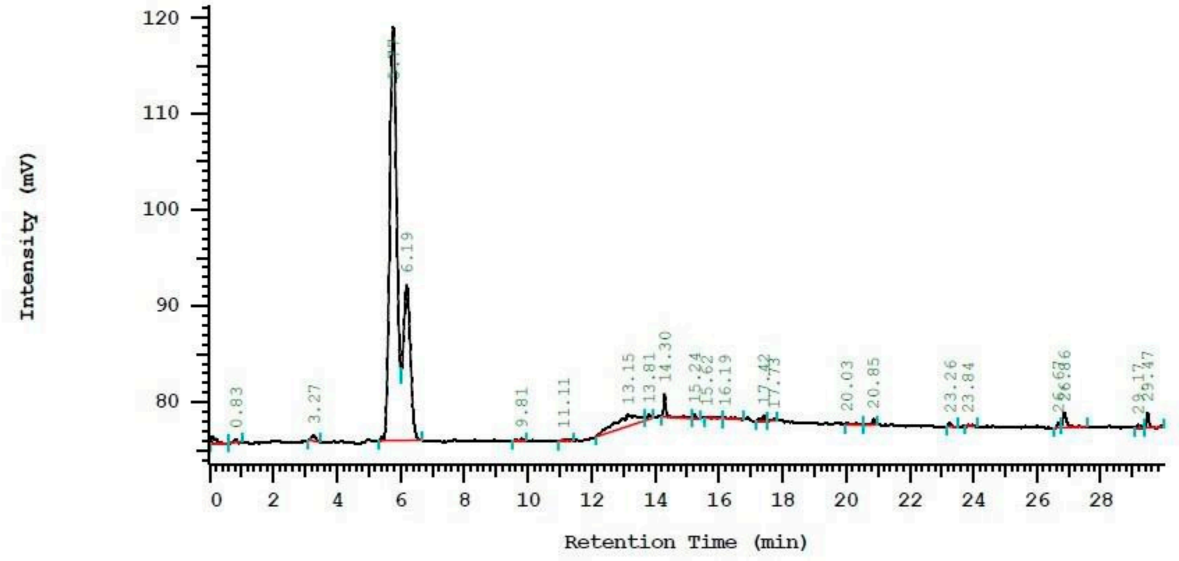

Figure S5. MRM chromatograms of BMAA, DAB, AEG, and D3BMAA

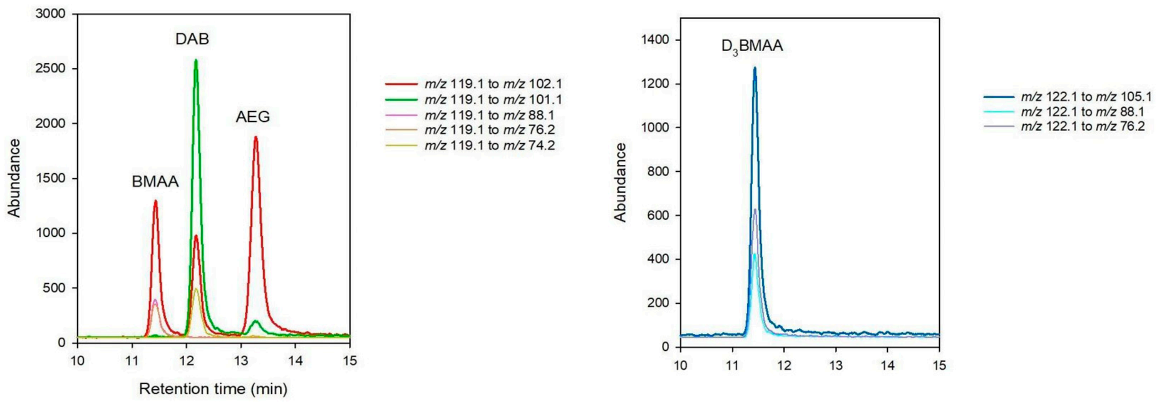

Supplement: Supplementary file 1 [file toxins-14-00215-s001.zip › Supplementary Material S2.pdf]
