# Supplementary material for: Assessment of Common Cyanotoxins in Cyanobacteria of Biological Loess Crusts
Source: Toxins (Basel). 2022 Mar 16;14(3):215. doi: 10.3390/toxins14030215 (PMC8953721; doi:10.3390/toxins14030215)

Supplementary Material S3. Raw gel images of PCR amplification of target genes in reference material and lack of amplification in a number of samples.

Figure S1. PCR amplification of *mcyE/nodF* genes in reference material. Primers used: HEPF, HEPR; Reference material: R1:NIES-107 (*Microcystis*), R2: PCC7820 (*Microcystis*), R3: PCC7806 (*Microcystis*).

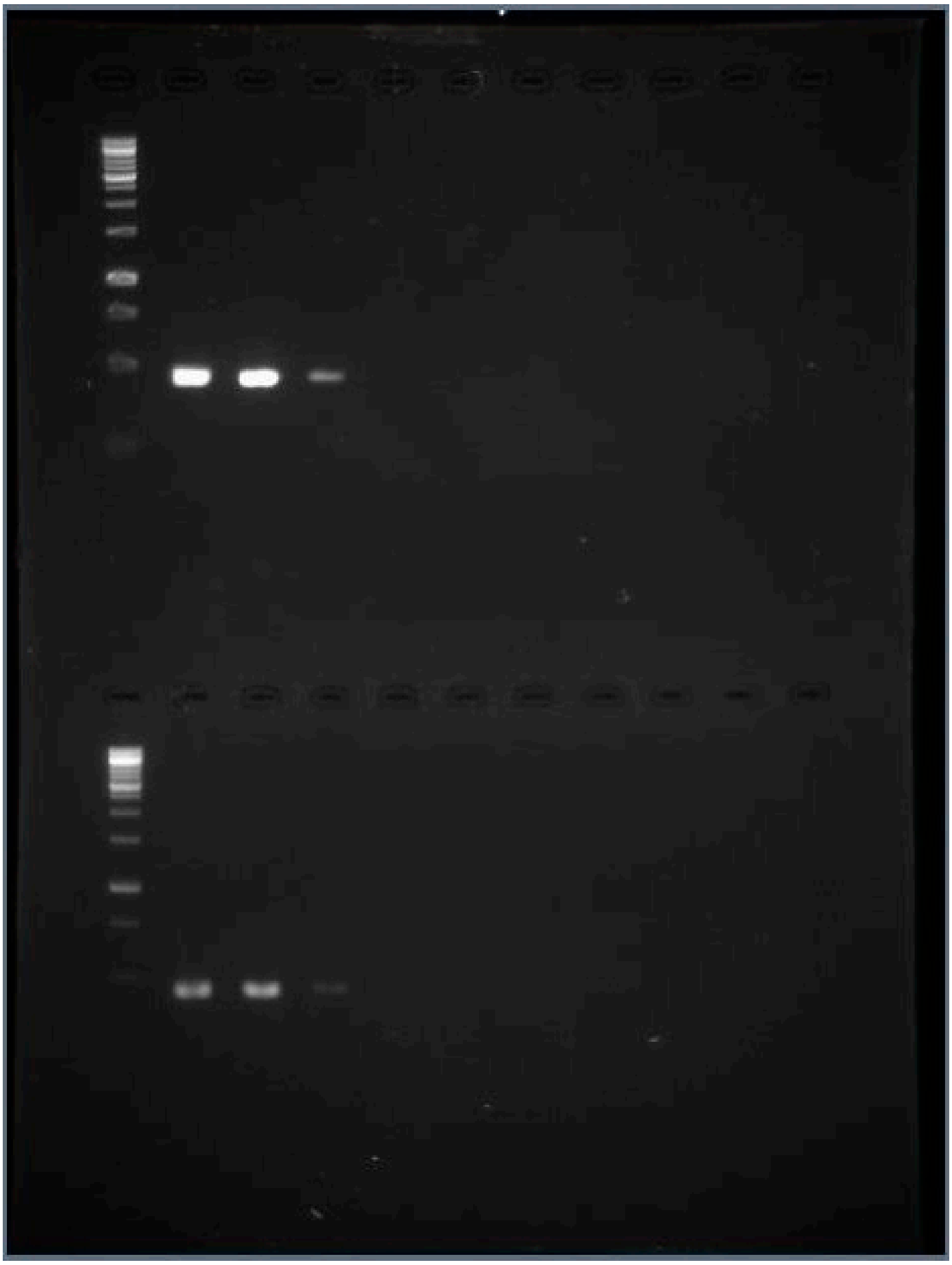

Figure S2. PCR amplification of the *cyrJ* gene in reference material. Primers used: cyrJ_F, cyrJ_R ; Reference material: R1: CS-505 (*Cylindrospermopsis*), R2: CS-506 (*Cylindrospermopsis*), R3: *Anabaena* 966.

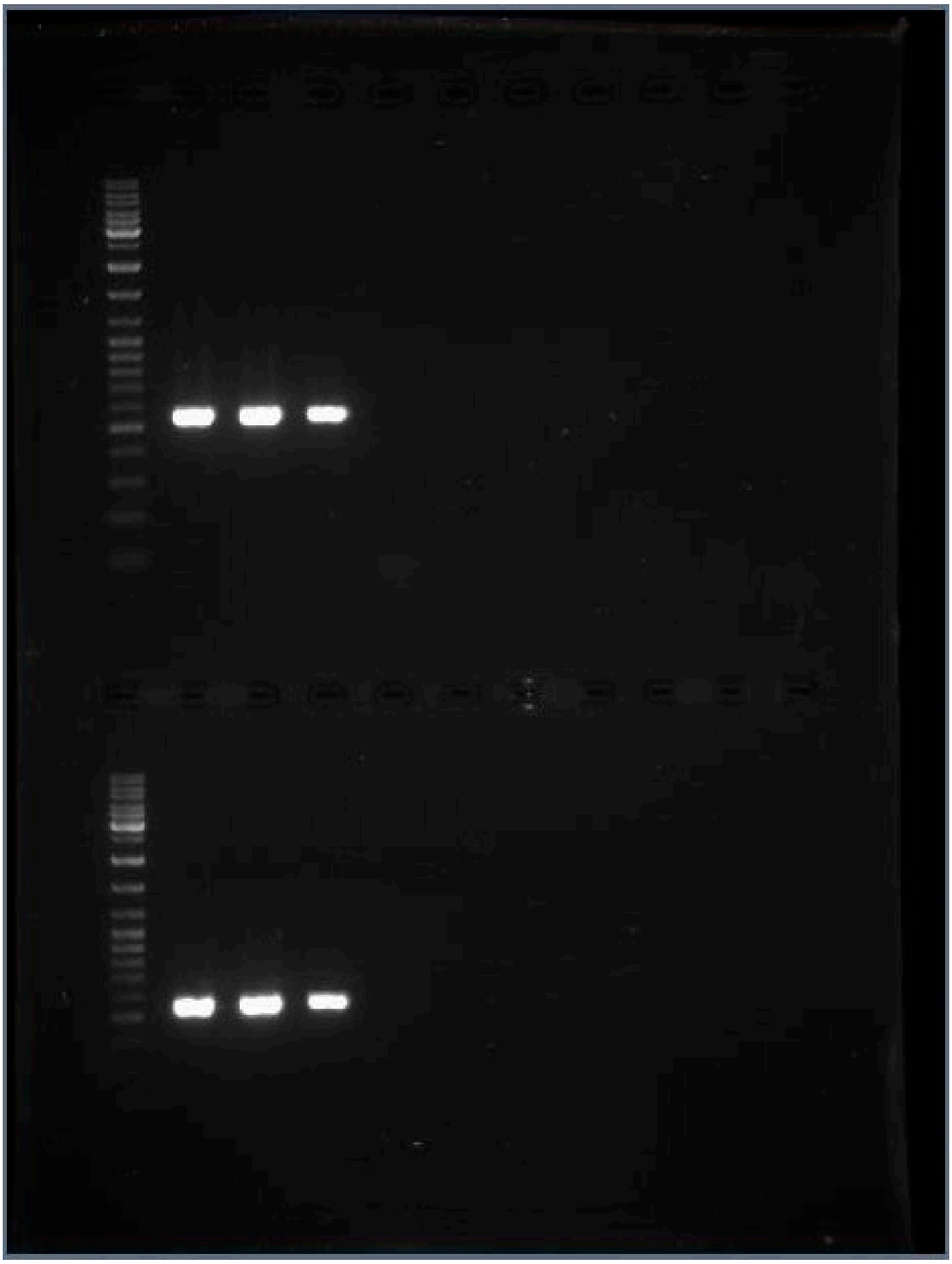

Figure S3. PCR amplification of *sxtA* gene in reference material. Primers used: stxA855_F, sxtA1480_R; Reference material: R1: CS-337/01 (*Dolichospermum*), R2: CS-537/13 (*Dolichospermum*).

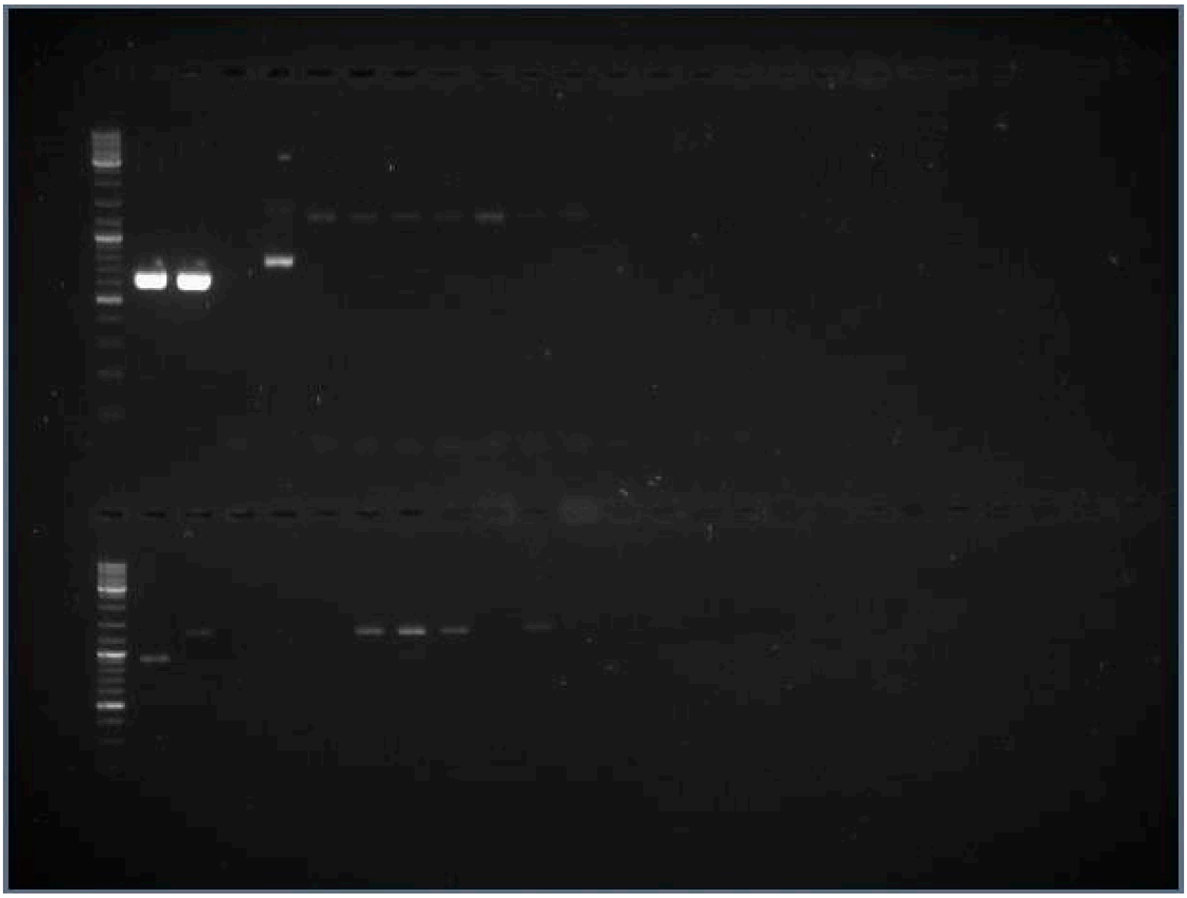

Figure S4. PCR amplification of *sxtG* gene in reference material. Primers used: sxtG432_F, sxtG928_R; Reference material: R1: CS-337/01 (*Dolichospermum*), R2: CS-537/13 (*Dolichospermum*).

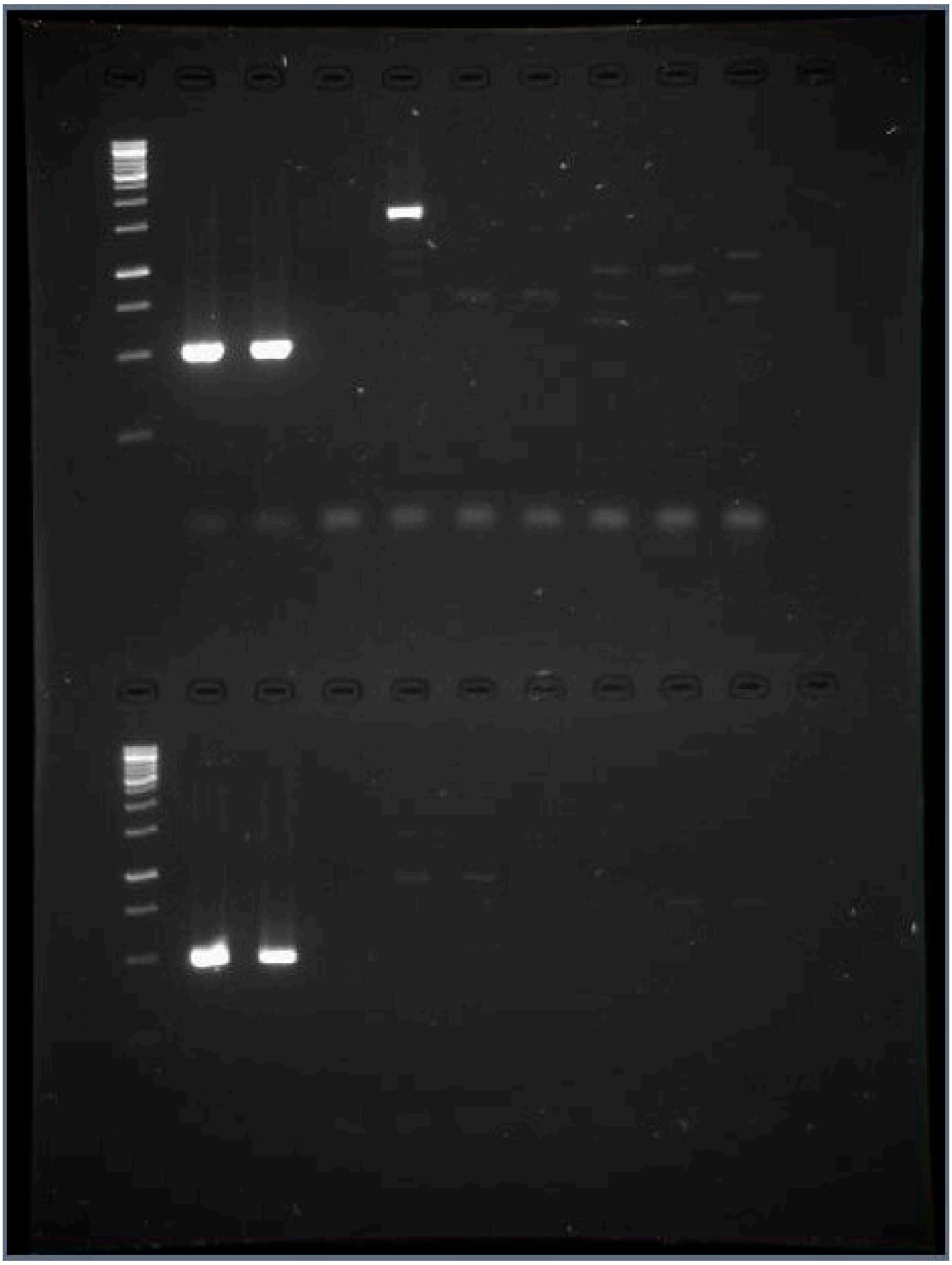

Figure S5. PCR amplification of *sxtS* gene in reference material. Primers used: sxtS205_F, sxtS566_R; Reference material: R1: CS-337/01 (*Dolichospermum*), R2: CS-537/13 (*Dolichospermum*).

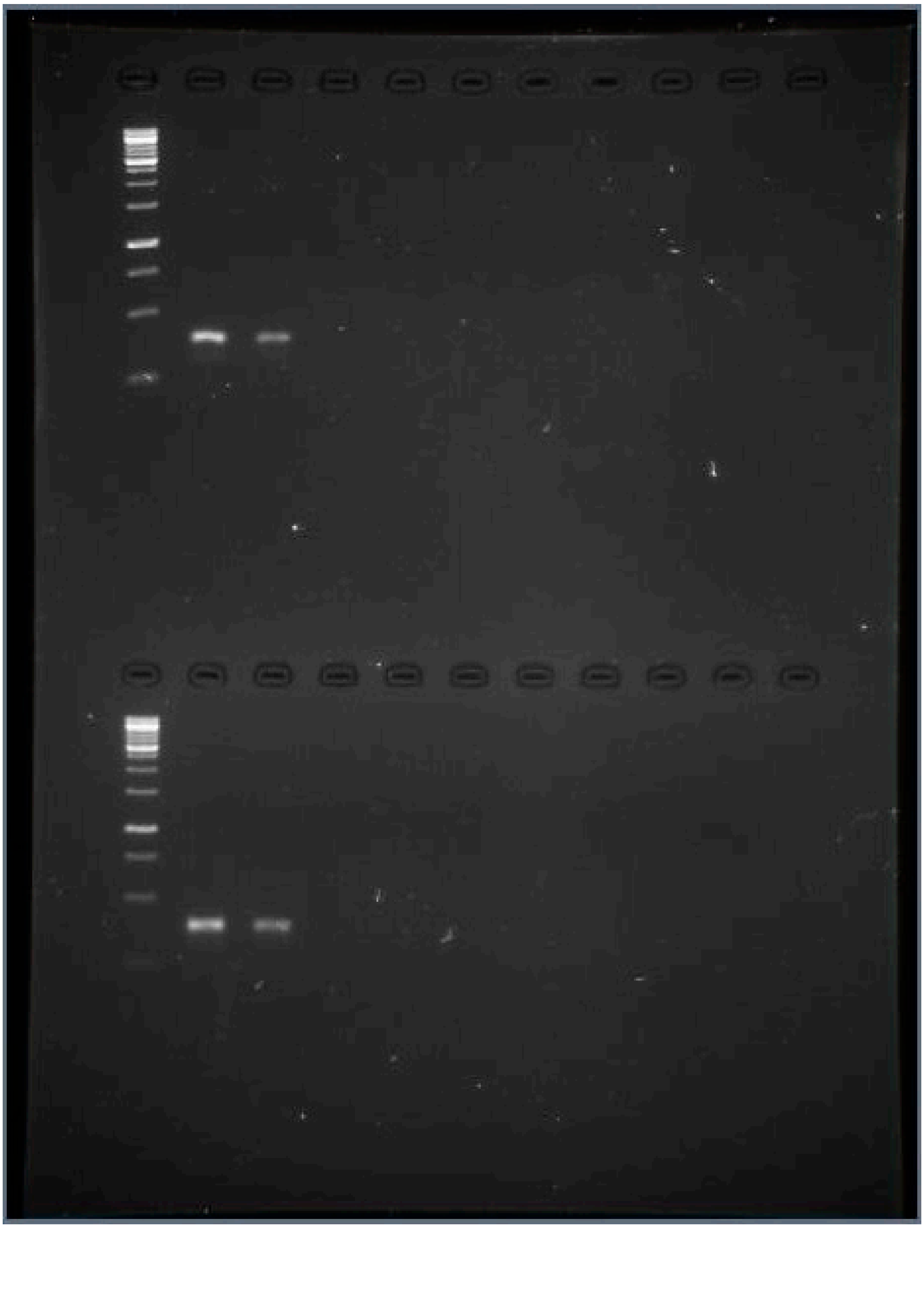

Figure S6. PCR amplification of *anaC* gene in reference material. Primers used: anaC-genF, anaC-genR; Reference material: R1: *Anabaena* 123

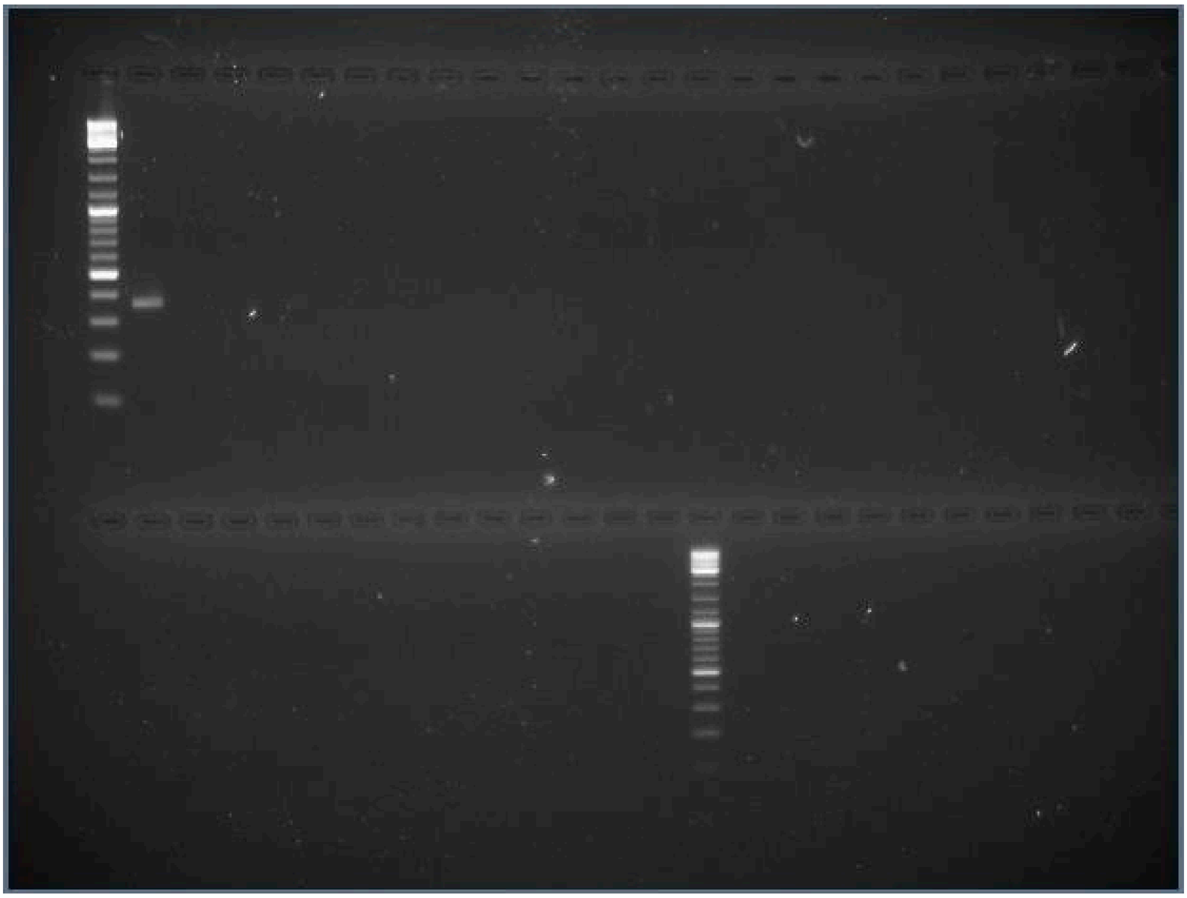

Supplement: Supplementary file 1 [file toxins-14-00215-s001.zip › Supplementary Material S3.pdf]
